# Targeting High Mobility Group Box 1 in Subarachnoid Hemorrhage: A Systematic Review

**DOI:** 10.3390/ijms21082709

**Published:** 2020-04-14

**Authors:** Sajjad Muhammad, Shafqat Rasul Chaudhry, Ulf Dietrich Kahlert, Martin Lehecka, Miikka Korja, Mika Niemelä, Daniel Hänggi

**Affiliations:** 1 Department of Neurosurgery, Heinrich-Heine University Medical Center, 40225 Düsseldorf, Germany; ulf.kahlert@med.uni-duesseldorf.de (U.D.K.); daniel.haenggi@med.uni-duesseldorf.de (D.H.); 2 Department of Neurosurgery, University of Helsinki and Helsinki University Hospital, 00014 Helsinki, Finland; martin.lehecka@hus.fi (M.L.); miikka.korja@hus.fi (M.K.); mika.niemela@hus.fi (M.N.); 3 Shifa College of Pharmaceutical Sciences, Shifa Tameer-e-Millat University, Islamabad 44000, Pakistan; shafqatrasul@yahoo.com

**Keywords:** subarachnoid hemorrhage, damage-associated molecular pattern molecules (DAMPs), alarmins, HMGB1 (High mobility group box 1), CVS (Cerebral vasospasm)

## Abstract

Aneurysmal subarachnoid hemorrhage (aSAH) is a complex and potentially deadly disease. Neurosurgical clipping or endovascular coiling can successfully obliterate ruptured aneurysms in almost every case. However, despite successful interventions, the clinical outcomes of aSAH patients are often poor. The reasons for poor outcomes are numerous, including cerebral vasospasm (CVS), post-hemorrhagic hydrocephalus, systemic infections and delayed cerebral ischemia. Although CVS with subsequent cerebral ischemia is one of the main contributors to brain damage after aSAH, little is known about the underlying molecular mechanisms of brain damage. This review emphasizes the importance of pharmacological interventions targeting high mobility group box 1 (HMGB1)-mediated brain damage after subarachnoid hemorrhage (SAH) and CVS. We searched Pubmed, Ovid medline and Scopus for “subarachnoid hemorrhage” in combination with “HMGB1”. Based on these criteria, a total of 31 articles were retrieved. After excluding duplicates and selecting the relevant references from the retrieved articles, eight publications were selected for the review of the pharmacological interventions targeting HMGB1 in SAH. Damaged central nervous system cells release damage-associated molecular pattern molecules (DAMPs) that are important for initiating, driving and sustaining the inflammatory response following an aSAH. The discussed evidence suggested that HMGB1, an important DAMP, contributes to brain damage during early brain injury and also to the development of CVS during the late phase. Different pharmacological interventions employing natural compounds with HMGB1-antagonizing activity, antibody targeting of HMGB1 or scavenging HMGB1 by soluble receptors for advanced glycation end products (sRAGE), have been shown to dampen the inflammation mediated brain damage and protect against CVS. The experimental data suggest that HMGB1 inhibition is a promising strategy to reduce aSAH-related brain damage and CVS. Clinical studies are needed to validate these findings that may lead to the development of potential treatment options that are much needed in aSAH.

## 1. Introduction

Aneurysmal subarachnoid hemorrhage (aSAH) is a devastating cerebrovascular event that accounts for only 5% of all stroke events. The mortality rate is approximately 50% (ranging from 32 to 67%) and affects patients of relatively younger age than the ischemic stroke, depriving society of potential productive age individuals [1]. Almost one third of the survivors have lifelong disabilities and cognitive problems [2]. In almost 85% of the cases, a rupture of the intracranial aneurysms causes a flooding of the subarachnoid space with the extravasated blood and can also even involve deeper brain parenchyma and ventricles [3]. Intracranial aneurysms are weakened lesions or abnormal dilatations that form in intracranial blood vessels, usually at arterial bifurcation sites, due to the shear stress of heightened blood flow pressure and are characterized by chronic inflammation [4]. A growing body of evidence has suggested that an upregulated inflammatory response in the arterial vessel walls underlies the development, growth and rupture of intracranial aneurysms [4]. Intracranial aneurysms usually occur at a frequency of 3–5% in the adult population with a slight prevalence in females [4]. While an obliteration of the aneurysm from the arterial circulation is achieved by micro-neurosurgical clipping or endovascular coiling in almost every case [5], the outcomes after aSAH remain poor. The poor outcomes are mainly related to post-aSAH complications such as cerebral vasospasm (CVS), hydrocephalus, seizures, delayed ischemic neurological deficits (DIND), cortical spreading depression, delayed cerebral ischemia (DCI), infections, cardiomyopathy and pulmonary edema [6]. Most complications occur within the first two weeks after the initial bleeding [7].

The molecular mechanisms behind post-aSAH complications are complex and are likely initiated at the time of early brain injury (EBI). EBI is a result of transient global ischemia that in turn occurs when blood transits through the ruptured aneurysm and causes increased intracranial pressure (ICP). Increased ICP leads to the release of damage-associated molecular pattern molecules (DAMPs) from damaged or stressed central nervous system cells [5,7,8]. The extravasated blood and its degradation products also cause damage to various cells in the immediate vicinity with a consequent release of DAMPs [9]. DAMP receptors are widely expressed in central nervous system cells, including endothelial cells, neurons, microglia, astrocytes and infiltrating immune cells. The interaction of DAMPs with receptors such as the receptor for advanced glycation end products (RAGE), the toll-like receptor (TLR)-2 and TLR-4, may initiate and drive the inflammatory response both in the brain parenchyma and cerebral blood vessels; hence, this links EBI with delayed inflammation. It is, therefore, important to identify the molecules that initiate and drive the immune response after aSAH.

High mobility group box 1 (HMGB1) is a classic prototypical protein DAMP that is ubiquitously expressed in the nucleus under normal conditions in all eukaryotic cells and facilitates transcription, but behaves as a DAMP once released from necrotic or inflammatory cells [10,11]. The HMGB1 molecule is encoded by around 10,000 base pairs on chromosome 13q12 in humans and is comprised of 214–216 amino acids constituting two DNA binding boxes, box A and box B, and an acidic tail [11,12]. HMGB1 has been known to bind several pattern recognition receptors such as TLR-2, TLR-4 and RAGE on immune cells to upregulate inflammation [9]. Consequently, HMGB1 has been shown to be an important mediator and pharmacological target in multiple inflammatory diseases, including sepsis, ischemia, trauma and arthritis [10,13,14]. Increasing evidence from experimental and clinical studies has suggested that HMGB1 might be an important target to reduce damage during EBI and life-threatening post-aSAH complications, including CVS. Here, we systematically reviewed the literature on the pharmacological targeting of HMGB1 in aSAH and the criteria for the selection of articles is presented in the Supplementary Materials (Figure S1).

## 2. Results

### 2.1. HMGB1 Is Released in Cerebrospinal Fluid (CSF) and Systemic Circulation after aSAH

DAMPs liberated upon damage to the cells of the CNS will ultimately find their way to the CSF as this fluid is immediately in contact with the CNS tissues. The release of HMGB1 into the cerebrospinal fluid (CSF) of patients after aSAH was first reported by Nakahara and colleagues in 2009. Interestingly, elevated HMGB1 levels were higher in the CSF of patients with poor clinical outcomes after aSAH. Moreover, HMGB1 levels were correlated with the tumor necrosis factor (TNF)-α, interleukin (IL)-6 and IL-8, suggesting an important role of HMGB1 in ongoing inflammation [15]. Extracellularly released HMGB1 is recognized by TLR-2, TLR-4, TLR-9, and RAGE [16] and leads to the induction of inflammatory signals, including nuclear factor kappa B (NF-κB). King and colleagues reported similar findings showing significant associations between CSF HMGB1 levels and aSAH severity (poorer Hunt and Hess grades) and disability among aSAH patients [17]. Furthermore, an experimental model of subarachnoid hemorrhage (SAH) confirmed HMGB1 upregulation and showed that HMGB1 was translocated into the cytosol of the microglia for active secretion [18]. More interestingly, elevated HMGB1 levels in systemic circulation were associated with CVS, poor functional outcomes and mortality one year after the aSAH, thus highlighting the prognostic potential of measuring plasma HMGB1 [19]. Moreover, HMGB1 levels measured in systemic circulation over a period of two weeks after the aSAH showed an association with CVS [20]. The elevated levels of HMGB1 were correlated with blood leukocytes and increased IL-6 levels [20]. Interestingly, HMGB1 levels measured on day 1 after the aSAH were also shown to correlate with elevated IL-10 levels later on day 7 after the aSAH [21]. Another clinical study described elevated CSF HMGB1 levels in acute hydrocephalus after the aSAH and strong correlations with different scores of aSAH severity, including Hunt and Hess grades, the World Federation of Neurological Surgeons (WFNS) score, and the Glasgow Coma Scale (GCS). Furthermore, HMGB1 levels were correlated with the duration of stay in the intensive care unit and poor outcomes after 3 months [22]. Wang and colleagues confirmed the association between CSF HMGB1 levels and poor outcomes after 3 months in a larger cohort of aSAH patients.

DCI mainly affects the clinical outcomes of aSAH patients and has been observed in approximately 30% of patients [23]. DCI is multifactorial and cerebral vasospasm is the primary cause leading to a DCI. A case series of SAH patients with DCI has shown a significant elevation of HMGB1 compared to the controls [24]. Moreover, the presence of minor allele G of rs2249825 is an independent predictor of DCI. Interestingly, this single nucleotide polymorphism of HMGB1 (C/G at 3814) leads to an enhanced HMGB1 expression and may consequently result in DCI [25].

### 2.2. Pharmacological Inhibition of HMGB1 Protects against Early Brain Injury after SAH

HMGB1 is released after the SAH and is associated with clinical outcomes and post-SAH complications. Thus, an evaluation of the blocking of the HMGB1 release during a SAH is warranted. Sun and colleagues observed an early release of HMGB1 (2 h) from neurons after the SAH [26]. An intraventricular injection of recombinant HMGB1 has been shown to upregulate inflammation as demonstrated by the upregulation of TLR-4, NF-κB, IL-1β and cleaved Caspase-3. Furthermore, the in vitro application of the red blood cell breakdown product hemoglobin (Hb) led to the upregulation and translocation of HMGB1 from the nucleus to the cytoplasm in neuronal cultures. Interestingly, the application of glycyrrhizic acid, a pharmacological inhibitor of HMGB1, downregulated IL-1β and thus, prevented the activation of glial cells [26]. Moreover, two other natural compounds, purpurogallin (a natural phenol) and 4′-*O*-β-d-glucosyl-5-*O*-methylvisamminol (4OGOMV), attenuated HMGB1 expression in a double hemorrhagic SAH rat model and effectively decreased CVS and CVS-related caliber changes in basilar arteries [27,28]. A similar study that employed rhinacanthin-C, an extract from *Rhinacanthus nasutus,* ameliorated SAH-associated increases in HMGB1 mRNA and protein levels, pro-inflammatory cytokines, cleavage of Caspase-3 and Caspase-9, and reduced apoptosis after SAH [29]. Resveratrol administration ameliorated the expression of HMGB1 along with other pro-inflammatory markers and reduced the brain edema, neuronal apoptosis, and improved neurological deficits at 24 h after the SAH [30]. Moreover, the increased expression of HMGB1 in vasospastic rat basilar arteries was observed at days 3, 5 and 7 after the SAH [31]. Li et al. have shown an increased basilar artery thickness and reduced luminal diameter with the increased expression of HMGB1 protein and mRNA of pro-inflammatory cytokines; these changes were ameliorated after glycyrrhizic acid supplementation for three days [32]. Glycyrrhizin supplementation has also been shown to downregulate the HMGB1 and pro-inflammatory markers’ (TNF-α, IL-1β) expression and improve neurological scores in a pre-chiasmatic SAH model [33]. Interestingly, HMGB1 expression and cytosolic translocation was inhibited by the Janus kinase 2 (JAK2)/signal transducer and activator of transcription 3 (STAT3) inhibitor AG490 and reduced brain edema, neuronal apoptosis, and improved neurological function after an experimental SAH [34].

Apoptosis, a form of programmed cell death, is implicated in SAH and the inhibition of apoptosis is associated with improved neurological deficits [5,8,35]. HMGB1 has been shown to activate apoptotic cascades in neurons and endothelial cells via the facilitation of proapoptotic p53 activation [36]. However, a programmed form of necrosis, called necroptosis, is characterized by the rupture of the cell with the extracellular release of DAMPs such as HMGB1. Intriguingly, receptor-interacting protein kinase-3 (RIPK-3)-mediated necroptosis in neurons was upregulated after an experimental SAH and was associated with an increased brain injury and cytosolic translocation of HMGB1 [35]. The inhibition of necroptosis by GSK’872, an inhibitor of RIPK-3, prevented cytosolic translocation and expression of HMGB1, and necroptosis, which was accompanied by reduced brain edema and improved neurological scoring [35].

Exosomes are nanovesicles secreted by almost all cells of the body and carry a diverse cargo consisting of proteins and different types of RNA and DNA, which play important roles in intercellular communication [36,37]. Exosomes derived from bone marrow mesenchymal stem cells (BMSCs) have been shown to alleviate the neurological deficits, brain edema and the blood–brain barrier disruption after an experimental SAH [36]. These BMSCs-derived exosomes reduced early brain injury by ameliorating the expression of pro-inflammatory molecules such as HMGB1, TLR-4 and TNF-α, and also reduced the proapoptotic p53 expression [36]. The beneficial effects of BMSCs-derived exosomes were demonstrated to stem from the increased expression of miRNA129-5p, which downregulated the inflammation mediated by the HMGB1–TLR-4 pathway during early brain injury [36].

### 2.3. Anti-HMGB1 Antibodies Confer Protection against CVS

**Figure 1:**
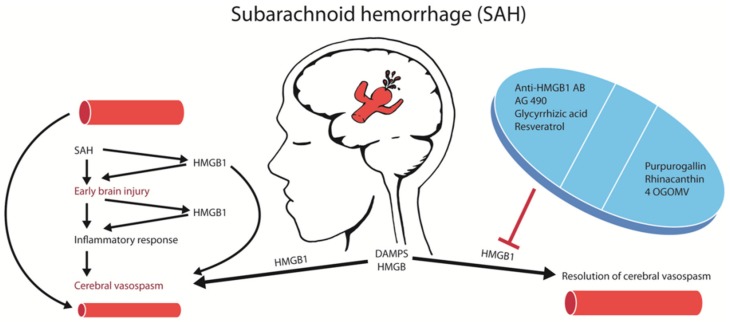
Strategies targeting high mobility group box 1 (HMGB1)-mediated brain damage after an aneurysmal subarachnoid hemorrhage (aSAH). Summary of the pharmacological strategies to block HMGB1 in SAH. Blocking HMGB1 with multiple agents reduced cerebral vasospasm and brain damage after SAH.

A more effective way to block HMGB1 is via neutralization with anti-HMGB1 antibodies. The administration of anti-HMGB1 antibodies in an experimental rat model of SAH decreased basilar artery vasospasm, extracellular translocation and the expression of HMGB1 in smooth muscle cells, as well as decreased the expression of contractile (endothelin type A (ET_A_)) receptor, angiotensin-II type 1 (AT_1_) receptor, protease activated receptor-1 (PAR1, thrombin receptor), thromboxane A_2_ (TXA2) receptor), inflammation-associated (TNF-α, IL-6, inducible nitric oxide synthase (iNOS), and TLR-4) molecules, plasma HMGB1 levels, improved the morphology and decreased the number of activated cerebral cortex microglia. Most importantly, the anti-HMGB1 antibodies reduced the delayed CVS (Figure 1) and decreased the severity of neurological deficits, such as the impairment of coordinated locomotor activity, which was assessed by an open field test with exposure to a novel environment [38]. Interestingly, the application of the anti-HMGB1 antibodies antagonized the sensitivity of the basilar arteries to vasoconstriction induced by the increasing doses of thrombin [38]. The anti-HMGB1 antibody treatment was effective in improving delayed CVS even if the antibody was applied 3 h after the SAH. These results demonstrate the therapeutic and translational potential of HMGB1 neutralization that can be achieved with delayed antibody treatment.

Another study aimed at the antibody-mediated antagonism of HMGB1 after SAH has shown that the prevention of vascular smooth muscle cell (VSMC) phenotypic switching and vascular remodeling may underlie the relieving of vasospasms [39]. The increased HMGB1 expression in the basilar artery was found to be associated with the overexpression of embryonic smooth muscle myosin heavy chain (Smemb) and osteopontin (OPN) and with a reduced expression of adult α-smooth muscle actin (α-SMA) and smooth muscle-myosin heavy chain (SM-MHC), but these changes were reversed by anti-HMGB1 antibodies [39]. Furthermore, anti-HMGB1 antibody administration was associated with an improvement in cortical blood flow, reduction in cerebral edema, cortical apoptosis, microglial activation and neurological impairments (assessed by modified Garcia scoring and beam balance test) [39]. Similarly to the aforementioned study, the anti-HMGB1 antibody also significantly reduced the expression of receptors for vasoconstrictive substances and reduced the threshold for a thrombin- or KCl-induced vasospasm in isolated basilar arteries after SAH [39]. Furthermore, authors demonstrated that the phenotypic and vascular remodeling reversal to partly depend upon PI3K/Akt activity [39]. These evidences indicate not only the reversal of vasospastic changes, but also the prevention against vascular remodeling and phenotypic switching, which result in the vessel wall thickening and reduced luminal diameters, which pave the way to delayed cerebral ischemia and neurological decline.

### 2.4. Subarachnoid Hemorrhage and Blood–Brain Barrier

The blood–brain barrier (BBB) allows the passage of selective substances into the brain, which are readily distributed to other body tissues. This specialized barrier consists of tightly sealed endothelial cells of the cerebral vasculature, which are further supported by pericytes and astrocytic endfeet at the abluminal side, thus limiting the direct access to the brain [40]. A great body of evidence suggests that an early disruption of the BBB takes place after ischemic strokes, hemorrhagic strokes and subarachnoid hemorrhages [41]. In an ischemia reperfusion stroke model, the BBB disruption was evident as early as 3 h after reperfusion, and it was consistent with the astrocytic endfeet swelling and detachment from the basement membranes and the disruption of endothelial tight junctions, as detected by electron microscopic examination [42]. Shi and colleagues have shown the dynamics of BBB disruption after ischemia-reperfusion injury in a mouse model of transient focal cerebral ischemia. An early breach of the BBB was evident within 30 min after reperfusion by a small tracer of about 3kDa and within 3 h by plasma IgG (of about 150 kDa), however, very large molecules of around 2000 kDa were evident only at 24 h post-ischemia-reperfusion [43]. Interestingly, the very early breach of the BBB was mediated by cytoskeletal rearrangements, leading to endothelial retraction, and later on by the disruption of the endothelial junctional proteins by matrix metalloproteinase-9 (MMP-9) [43]. A great body of evidence suggests that the increased levels of HMGB1 in the CSF and in the systemic circulation, after ischemic and hemorrhagic strokes, are due to the release of HMGB1 from damaged cells that gains entry into these compartments through the disrupted BBB. BBB impairment is more severe in the ischemic part of the brain [10]. Anti-HMGB1 antibody targeting HMGB1 prevented the BBB disruption, probably by reducing the expression of pro-inflammatory molecules and inhibiting the activation of glial cells [42]. Apart from anti-HMGB1 antibodies, the HMGB1 can be antagonized by glycyrrhizic acid, HMGB Box A, soluble RAGE (sRAGE), ethyl pyruvate and recombinant thrombomodulin. After aSAH, it has been shown that the early brain injury caused due to cerebral edema, which stems from early BBB disruption, leads to a poor clinical outcome [44]. The BBB disruption has been shown to be reduced after antagonizing HMGB1 and reducing cerebral edema in SAH animal models [39].

### 2.5. Soluble RAGE Protected against EBI

The multiligand receptor RAGE also exists as a soluble receptor form that results from the proteolytic cleavage of membranous RAGE lacking intracellular domains, or through alternate splicing [45]. Soluble RAGE acts as a competitive inhibitor of RAGE by interacting with its cognate ligands and thus, culminates and limits RAGE-mediated cellular signaling, cellular dysfunction and damage [45]. Consequently, soluble RAGE (sRAGE) can be used to sequester HMGB1. RAGE expression can be upregulated by its ligands such as HMGB1 in a positive feedback loop [46]. Both HMGB1 and its receptor RAGE are upregulated in the brain after aSAH. The expression of RAGE was particularly upregulated on neurons and microglia after the experimental SAH in rats and was correlated with the increased expression of p65, suggesting a main role of RAGE in mediating inflammation [47]. Consequently, the application of post-SAH CSF from patients or rats induced the RAGE expression and reduced the viability of neuronal cultures. Interestingly, the administration of a recombinant soluble form of RAGE to interfere with HMGB1 signaling reduced neuronal cell death both in vitro and in vivo [48]. These data are consistent with a previously described finding suggesting that interference with the HMGB1-signaling pathway protects against EBI. Table 1 (below) summarizes all the experimental agents employed to neutralize the effects of HMGB1 in different animal models of SAH.

## 3. Discussion

HMGB1 is a highly conserved non-histone nuclear protein and an important prototypical protein DAMP that is released both in the CSF and the systemic circulation in clinical and experimental SAH. The interaction between HMGB1 and pattern recognition receptors leads to the activation of downstream signaling pathways (including NF-κB pathways) and consequently to the expression of multiple pro-inflammatory genes.

The treatment of CVS via smooth muscle relaxants, such as calcium antagonists, endothelin receptor antagonists, and Rho-kinase inhibitors, does not significantly improve clinical outcomes. The lack of beneficial effects of smooth muscle relaxants is due to the complex pathophysiology of SAH, where the causal relation to the development of a delayed CVS is poorly understood. Both brain and vascular inflammation are closely related to the development of EBI and a delayed CVS [49]. Recent experimental studies have shown that HMGB1 is released from vascular smooth muscle cells and intracranial vessel walls, which may be the source of circulating HMGB1 as suggested in some clinical studies [9]. A causal relationship between the HMGB1 release and CVS has been established with in vitro experiments showing the reversal of a spastic vascular phenotype after treatment with anti-HMGB1 antibodies [38]. The release of HMGB1 may induce the expression of pro-inflammatory cytokines and vasoconstriction-inducing receptors, including PAR-1, TXA2 receptor, AT1 receptor and the ET_A_ receptor via interactions with pattern recognition receptors [38]. As an upstream event, the HMGB1 release during an EBI could theoretically be an excellent target for the treatment of both the EBI and the delayed CVS. Indeed, targeting HMGB1 with monoclonal antibodies or with pharmacological agents have reversed the delayed CVS (Figure 1) in animal SAH models [38].

HMGB1 effects could be mediated by multiple receptors, including TLR-4, TLR-2 and RAGE. Interestingly, these receptors have been shown to be involved in the inflammatory response after the SAH [50,51,52]. For instance, the HMGB1 ligation of the TLR-4 has been shown to activate MMP-9 (Matrix metalloproteinase-9), which contributes to early brain injury after an experimental SAH [53]. Nevertheless, further investigations are needed to establish the exact receptor pathway that is involved in the induction of delayed CVS. Intriguingly, on one hand the HMGB1 ligation of RAGE on monocytes/macrophages has been shown to enhance the ischemic brain damage, and on the other hand, HMGB1-signaling via RAGE drives an IL-10 release from M2-like macrophages (the anti-inflammatory phenotype of macrophages). In line with this notion, serum HMGB1 levels measured within 24 h after the aSAH showed a correlation with a latter increase in serum IL-10 levels measured on day 7 after the aSAH [21]. It is well known that anti-inflammatory mechanisms also upregulate in parallel to pro-inflammatory mechanisms to limit the damage, however, it would be interesting to evaluate further how these pro-inflammatory mechanisms are dominated by anti-inflammatory mechanisms, and how they contribute towards different post-aSAH complications and clinical outcomes, as several lines of evidence also report immunodepression after aSAH [21,54].

Anti-HMGB1 antibody treatment blocked the expression of pro-inflammatory cytokines (including IL-6, TNF, and TLR-4, iNOS) and vasoconstriction-inducing receptors, and reversed the contractile phenotype of the basilar artery and improved neurological outcomes [38]. Furthermore, HMGB1 has been implicated in vascular smooth muscle cell phenotype switching and vascular remodeling, which may underlie the thickened vascular walls along with the reduced intraluminal diameter [39]. These changes ultimately lead to cerebral ischemia and neurological deficits [39]. These results suggest that targeting HMGB1 may be a better option to treat delayed CVS than simply with smooth muscle relaxants as previously done. Another possible mechanism of inflammation-mediated delayed CVS is the expression of COX-2 in the vasculature. COX-2 is a target gene of NF-κB that can be activated by HMGB1 via pattern recognition receptors. Thus, a continuous mobilization of HMGB1 from cerebral vessels, starting early after the aSAH, leads to the expression of pro-inflammatory cytokines and receptors that in turn mediate the CVS. Anti-HMGB1 antibodies are perhaps a new approach to interrupt this cascade of events and to induce a relaxed phenotype of smooth muscle cells, and consequently reduce the CVS and improve the clinical outcomes.

Interestingly, different isoforms of HMGB1 exist after the extracellular release with distinct functionalities and differences in their interactions with various receptors [20,55]. These differences owe to the redox states of three cysteine residues (C23, C45 and C106) in the Box B of the HMGB1 molecule. For instance, when all of these cysteine residues are in a reduced thiolated state, HMGB1 activates RAGE and promotes CXCL12/CXCR4 signaling [55]; whereas the oxidized form of HMGB1 with disulfide linkages between C23–C45 has a greater propensity to activate TLR-2 and TLR-4 to strongly upregulate inflammation [55]. Furthermore, a fully oxidized and sulphonated form is seen during inflammation resolution and is inert [20]. Intriguingly, an oxidized form of HMGB1 has been shown to play a neuroprotective role during the recovery phase of the SAH (day 14 after the SAH), depicted by an inability to stimulate serum and CSF TNF-α upsurge and enhancing neurotrophin expression, as opposed to a reduced form of HMGB1 [56]. Furthermore, the inhibition of HMGB1 and RAGE signaling during this delayed recovery phase after the SAH (day 14 after the SAH) was associated with a decline in the neurotrophic growth factors (Nerve growth factor (NGF), Brain derived neurotrophic factor (BDNF), vascular endothelial growth factor (VEGF)) and a reduction in neurogenesis as assessed by BrdU and DCX positive neurons [56]. The inhibition of RAGE by FPS-ZM1 and HMGB1 by ethyl pyruvate and glycyrrhizin also enhanced brain water content and the functional neurological impairment during this delayed recovery phase after SAH [56]. It would be quite interesting to study the dynamics of these distinct isoforms over the course of early brain injury and CVS after the SAH, and the impact of modulating these HMGB1 isoforms on inflammatory changes and neurological function after an experimental SAH. As mentioned earlier, HMGB1–RAGE signaling in macrophages has been shown to enhance ischemic brain damage as well as the secretion of anti-inflammatory cytokine (IL-10 secreted by M2-type macrophages). It would be also quite interesting to elucidate the dynamics of the structurally and functionally different isoforms of HMGB1 over the entire course of early and delayed brain injury in SAH patients, and how they modulate the activity of macrophages involving RAGE and other cognate receptors.

HMGB1 also contributes to coagulation, as depicted by the platelets’ aggregation upon the ligation of RAGE, platelet activation and thrombus formation due to HMGB1/TLR-4 signaling and the enhanced expression of tissue factor in monocytes and endothelial cells [57]. Intriguingly, during an early brain injury after SAH, there is also evidence of microvasospasms and microthrombosis and the degree of arteriolar constriction correlates with microthrombotic frequency [58]. These mechanisms could compromise blood flow independently of cerebral perfusion pressure [58]. Furthermore, Clazosentan was also found to be ineffective towards relieving these microvasospasms and improving deficits in the experimental SAH rat model [59]. Previously, a failure of Clazosentan to improve the clinical outcomes of SAH patients led to a renewed interest in exploring the additional mechanisms of brain injury, other than angiographic vasospasm [3]. It might be a new beginning to explore the underpinnings of microvasospasms and exploring the role of inflammation, as inflammation and thrombosis cannot be segregated into distinct events independent of each other. It would be interesting to study the implications of HMGB1 and its cognate receptors in the microvasospasms and microthrombosis and the impact of modulating HMGB1 on the inflammation underlying these events. Thrombomodulin has also been shown to scavenge HMGB1 [60] and it may be employed to study its impact on CVS, microthrobosis and microvasospasms during EBI after aSAH. Altogether, the aforementioned pieces of evidence suggest an indispensable role of HMGB1 after an aSAH and its contribution to aSAH-led complications, especially CVS and poor neurological outcomes. Despite the shift in the traditional paradigm, i.e., from CVS leads to DCI and poor outcomes, towards a complex multifactorial pathophysiology involving varied contributions from EBI, cortical spreading depression and inflammation, there is still a population of CVS patients who develop DCI and poor outcomes [61]. Furthermore, inflammation is associated with EBI, CVS, DCI and poor outcomes and the inhibition of HMGB1-mediated inflammation could be promising to benefit aSAH patients at increased risk of these complications and poor outcomes. 

Due to the presence of an intact blood–brain barrier, pharmacological agents have limited excess to the brain. However, during pathological conditions including cerebral ischemia or subarachnoid hemorrhage, disrupted blood–brain barrier facilitates the access of pharmacological agents to the injured brain. Although there are differences in different rodent SAH models and also differences of the BBB among the various animals used for the SAH, the ischemic insult induces a massive disruption of the BBB [40,41,62]. Furthermore, the observance of the pharmacological effects of anti-HMGB1 antibodies at the CNS level also suggest their access through the permeable BBB. Reduction in the permeability of the BBB after the administration of anti-HMGB1 antibodies may also argue against any further access of these antibodies through the BBB, but may also reflect the culmination of the effects which may no longer be required, suggesting healing based self-termination of the CNS effects. However, pharmacokinetics of anti-HMGB1 antibodies and other anti-HMGB1 molecules remain to be elucidated in these SAH animal models. Recently, radiolabeled antibodies have been imaged to quantify the penetration through the blood–brain barrier. It is also known that antibody penetration across the BBB is more into the brain areas which are severely affected by tumors [63]. However, as mentioned above, strokes may lead to the massive disruption of the BBB and interestingly, ischemic brain regions have also shown greater penetration through the BBB [10]. Glycyrrhizic acid has been evaluated as an adjunctive anti-inflammatory agent to treat depression in a randomized-placebo-controlled clinical trial [64], but similar studies in aSAH could be possible. There is a need to characterize the pharmacokinetics and bioavailability of the drugs at the CNS level (e.g., in the CSF), particularly in patients who underwent extraventricular drain placement. Altogether, the experimental strategies antagonizing HMGB1 await clinical translation to benefit aSAH-afflicted patients by improving their outcomes, as pharmacological interventions in the context of aSAH are still scarce.

## 4. Conclusions

The experimental data suggest that HMGB1 inhibition is a promising strategy to reduce SAH-related brain damage and CVS. The brain accessibility of pharmacological agents targeting HMGB1 need to be evaluated in detail. Clinical studies are needed to validate this strategy in humans, and aid in the development of potential treatment options that are much needed in aSAH.

## Figures and Tables

**Table 1:** HMGB1 as a drug target in Subarachnoid Hemorrhage.

Sr. No.	Author/Year	Study Type (Animal Models)	HMGB1 Inhibitor (ip/ICV)	Dose	Key Results
1	An et al. 2018 [34]	Rat Endovascular perforation model	AG 490	5 mM in 2 mL DMSO, ICV 30 min before SAH	Reduced apoptosis, edema, improved neurological score
2	Ieong et al. 2018 [33]	Rat Pre-chiasmatic hemorrhage model	Glycyrrhizin	15 mg/Kg after SAH, 6 h, 12 h, 18 h, ip	Reduced apoptosis, edema, improved neurological score
3	Li et al. 2017 [32]	Rat SAH model Double hemorrhage	Glycyrrhizic acid	10 mg/Kg OD for 3 days, ip	Improved neurologic function, prevented CVS and inflammatory cytokines expression
4	Zhang et al. 2016 [30]	Pre-chiasmatic hemorrhage model	Resveratrol	60 mg/Kg in 1% DMSO 2 h and 12 h after SAH, ip	Reduced apoptosis, edema, neurological impairment
5	Chang et al. 2015 [28]	Rat SAH model Double hemorrhage	4OGOMV	100/200/400 µg/Kg/day starting 1 h post SAH for 7 days through mini osmotic pump	Improved CVS, neurological deficits, reduced expression of inflammatory mediators and neuronal apoptosis
6	Haruma et al. 2016 [38]	Rat SAH model Single hemorrhage	Anti-HMGB1 Antibody	mAb (IgG2a) 1 mg/Kg twice with 24 h interval, iv	Improved CVS, neurological deficits, reduced expression of inflammatory mediators and receptors for vasospastic mediators
7	Chang et al. 2016 [29]	Rat SAH model Double hemorrhage	Rhinacanthin	100/200/400 µmol/Kg/day orally in corn oil starting at 1 h after SAH	Reduced apoptosis, improved CVS, neurological deficits, reduced inflammatory mediator expression
8	Chang et al. 2014 [27]	Rat SAH model Double hemorrhage	Purpurogallin	100/200/400 µg/Kg/day starting 1 h after SAH through mini osmotic pumps for 5 days	Reduced CVS, inflammatory mediators expression and improved neurological deficits
9	Wang et al. 2019 [39]	Rat Endovascular perforation model	Anti-HMGB1 Antibody	mAb 1 mg/Kg twice with a 24 h interval after SAH, iv	Reduced CVS, VSMCs phenotype switching & remodelling, brain edema, apoptosis, neurological deficits
10	Chen et al. 2018 [35]	Rat Endovascular perforation model	GSK 872	6 µL of 25 mM GSK 872 in 1% DMSO after 30 min of SAH, ICV	Reduced brain edema, improved neurological scores and reduced neuronal necroptosis
11	Xiong et al., 2020 [36]	Rat Endovascular perforation model	BMSCs derived exosomes	1 h after SAH, 200 µg of MSCs-Exo and PBS to final volume of 200 µL, iv	Reduced neurological deficits, brain edema, BBB permeability, mortality, apoptosis and inflammation

ip: intraperitoneal; ICV: intracerebroventricular; iv: intravenously; BMSCs: bone marrow derived mesenchymal stem cells; BBB: blood–brain barrier; DMSO: dimethylsulfoxide; Exo: exosomes; CVS: cerebral vasospasm; VSMC: vascular smooth muscle cells; PBS: phosphate buffered saline.

## Supplementary Materials

Supplementary materials can be found at https://www.mdpi.com/1422-0067/21/8/2709/s1. Figure S1: Schematic representation of the literature search.
