# Supplementary material for: Targeting High Mobility Group Box 1 in Subarachnoid Hemorrhage: A Systematic Review
Source: Int J Mol Sci. 2020 Apr 14;21(8):2709. doi: 10.3390/ijms21082709 (PMC7215307; doi:10.3390/ijms21082709)
Supplement: Supplementary file 1 [file ijms-21-02709-s001.pdf]

International Journal of *Molecular Sciences*

MDPI

Supplementary Materials

Targeting High Mobility Group Box 1 in Subarachnoid Hemorrhage: A Systematic Review

Sajjad Muhammad [1,3,*], Shafqat Rasul Chaudhry [2], Ulf Dietrich Kahlert [1], Martin Lehecka [3], Miikka Korja [3], Mika Niemelä [3] and Daniel Hänggi [1]

[1] Department of Neurosurgery, Heinrich-Heine University Medical Center, Düsseldorf, Germany; ulf.kahlert@med.uni-duesseldorf.de (U.D.K.); daniel.haenggi@med.uni-duesseldorf.de (D.H.)
[2] Shifa College of Pharmaceutical Sciences, Shifa Tameer-e-Millat University, Islamabad, Pakistan; shafqatrasul@yahoo.com
[3] Department of Neurosurgery, University of Helsinki and Helsinki University Hospital, Helsinki, Finland; martin.lehecka@hus.fi (M.L.); miikka.korja@hus.fi (M.K.); mika.niemela@hus.fi (M.N.)
* Correspondence: sajjad.muhammad@med.uni-duesseldorf.de; Tel.: +49-15168460755

Methodology

For retrieval of potential articles to be included in this review, we searched Pubmed, Ovid medline, and Scopus using "Subarachnoid hemorrhage" as a MeSH term in combination with HMGB1 in any field. Based on these criteria, initially a total of thirty one (31) articles were retrieved. After excluding the duplicates and selecting the relevant references from the retrieved articles, eleven publications were finally selected to review the pharmacological interventions targeting HMGB1 in SAH. An outline of the selection of the literature regarding pharmacological interventions targeting HMGB1 in SAH is represented in Figure 1.

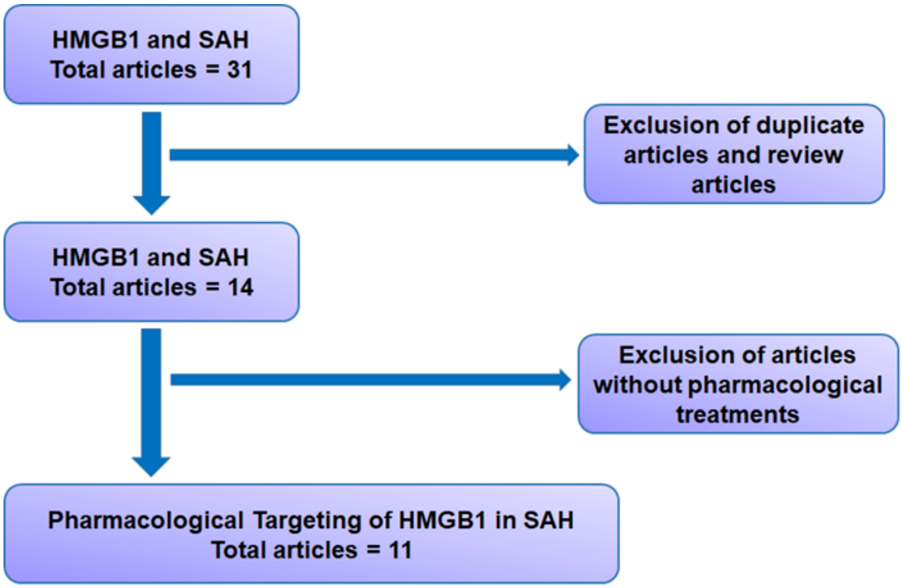

Figure 1. Schematic representation of the literature search. Schematic representation of literature search for articles on pharmacological interventions targeting HMGB1 in experimental SAH. Pubmed, Ovid medline, and Scopus were searched for "Subarachnoid Hemorrhage" as MeSH term and "HMGB1" in any field to retrieve the potential articles for this review.
